# Serotonin transporter knockout in rats reduces beta- and gamma-band functional connectivity between the orbitofrontal cortex and amygdala during auditory discrimination

**DOI:** 10.1093/cercor/bhae334

**Published:** 2024-08-11

**Authors:** Morgane Boillot, Jordi ter Horst, José Rey López, Ilaria Di Fazio, Indra L M Steens, Michael X Cohen, Judith R Homberg

**Affiliations:** Department of Cognitive Neuroscience, Radboud University Medical Center, Donders Institute for Brain, Cognition and Behaviour, Kapittelweg 29, 6525 EN, Nijmegen, Netherlands; Department of Cognitive Neuroscience, Radboud University Medical Center, Donders Institute for Brain, Cognition and Behaviour, Kapittelweg 29, 6525 EN, Nijmegen, Netherlands; Department of Cognitive Neuroscience, Radboud University Medical Center, Donders Institute for Brain, Cognition and Behaviour, Kapittelweg 29, 6525 EN, Nijmegen, Netherlands; Department of Cognitive Neuroscience, Radboud University Medical Center, Donders Institute for Brain, Cognition and Behaviour, Kapittelweg 29, 6525 EN, Nijmegen, Netherlands; Department of Cognitive Neuroscience, Radboud University Medical Center, Donders Institute for Brain, Cognition and Behaviour, Kapittelweg 29, 6525 EN, Nijmegen, Netherlands; Department of Cognitive Neuroscience, Radboud University Medical Center, Donders Institute for Brain, Cognition and Behaviour, Kapittelweg 29, 6525 EN, Nijmegen, Netherlands; Department of Cognitive Neuroscience, Radboud University Medical Center, Donders Institute for Brain, Cognition and Behaviour, Kapittelweg 29, 6525 EN, Nijmegen, Netherlands

**Keywords:** amygdala, local field potential, orbitofrontal cortex, reward, serotonin transporter knockout

## Abstract

The orbitofrontal cortex and amygdala collaborate in outcome-guided decision-making through reciprocal projections. While serotonin transporter knockout (SERT^−/−^) rodents show changes in outcome-guided decision-making, and in orbitofrontal cortex and amygdala neuronal activity, it remains unclear whether SERT genotype modulates orbitofrontal cortex–amygdala synchronization. We trained SERT^−/−^ and SERT^+/+^ male rats to execute a task requiring to discriminate between two auditory stimuli, one predictive of a reward (CS+) and the other not (CS−), by responding through nose pokes in opposite-side ports. Overall, task acquisition was not influenced by genotype. Next, we simultaneously recorded local field potentials in the orbitofrontal cortex and amygdala of both hemispheres while the rats performed the task. Behaviorally, SERT^−/−^ rats showed a nonsignificant trend for more accurate responses to the CS−. Electrophysiologically, orbitofrontal cortex—amygdala synchronization in the beta and gamma frequency bands during response selection was significantly reduced and associated with decreased hubness and clustering coefficient in both regions in SERT^−/−^ rats compared to SERT^+/+^ rats. Conversely, theta synchronization at the time of behavioral response in the port associated with reward was similar in both genotypes. Together, our findings reveal the modulation by SERT genotype of the orbitofrontal cortex—amygdala functional connectivity during an auditory discrimination task.

## Introduction

Effective decision-making requires accurate anticipation of outcomes based on prior experience and the ability to learn new associations and adapt behavior to the constantly changing environment (O’Doherty et al. 2017). The orbitofrontal cortex (OFC) and the amygdala (AMY), particularly the basolateral amygdala (BLA), are crucial for outcome-guided learning and decision-making (Schoenbaum et al. 1998). They have distinct yet intricate functions supported by their reciprocal monosynaptic projections. BLA neurons encode evolving outcome–value representations and signal attention to salient outcomes. The OFC utilizes this information to generate integrated cue-triggered task representations in distinct neuronal ensembles, enabling adaptive predictions about future outcomes (for reviews, see Bissonette and Roesch 2015; Wassum and Izquierdo 2015; Sharpe and Schoenbaum 2016; Takehara-Nishiuchi 2022). The OFC and AMY are interdependent and collaborate in reward-based decision-making, as shown with disconnecting lesions in monkeys and rodents (Baxter et al. 2000; Zeeb and Winstanley 2013; Lucantonio et al. 2015; Fiuzat et al. 2017) and more recently with chemogenetic or optogenetic interventions targeting projection neurons between the medial or lateral OFC and the BLA (Lichtenberg et al. 2017; Groman et al. 2019; Malvaez et al. 2019; Lichtenberg et al. 2021; Sias et al. 2021).

The OFC and AMY both receive dense serotonergic input predominantly from the dorsal raphe serotonin (5-HT) neurons (Ren et al. 2018), which encode the anticipation and reception of rewarding and aversive outcomes through tonic and phasic changes in their firing rate (Liu et al. 2014; Cohen et al. 2015; Li et al. 2016). The serotonin transporter (SERT or 5-HTT) regulates the synaptic 5-HT concentration by reuptake into the presynaptic neuron. Accordingly, it was demonstrated that SERT knockout (SERT^−/−^) rodents displayed elevated extracellular 5-HT concentration, both in basal conditions and evoked by electrical stimulation, the latter being insensitive to the stimulation frequency (Homberg et al. 2007; Jennings et al. 2010). This suggests that extracellular 5-HT concentration is not only increased but might also not follow variations in serotonergic neurons’ firing rates in SERT^−/−^ animals.

Various studies have shown that SERT knockout in rodents also influences outcome-guided behavior. For example, reversal learning, in which previously learned contingencies between conditioned stimuli (CS) and rewarding or neutral outcomes are reversed, is accelerated in SERT knockout (SERT^−/−^) rodents (Brigman et al. 2010; Nonkes et al. 2013). SERT^−/−^ rats are also less able to update their behavioral response to the CS after reward devaluation (Nonkes et al. 2010). In a discriminative fear learning task, SERT^−/−^ mice learnt the associations with the respective CS, i.e. a fear-inducing CS predicting foot shock (CS+) and a neutral CS never associated with foot shock (CS−), faster than their wildtype counterparts (SERT^+/+^ mice) (Lima et al. 2019).

Brain regions communicate through neural oscillations. The study by Lima et al. (2019) was among the few to date to have investigated the effect of SERT genotype on local or interareal network activity via local field potential (LFP) recordings in aversive learning. SERT^−/−^ mice showed stronger theta frequency oscillations in the AMY specifically during CS+ presentations compared to SERT^+/+^ mice. In another study, SERT^−/−^ mice displayed increased theta synchronization between the AMY and medial prefrontal cortex during recall of fear extinction (Narayanan et al. 2011).

By contrast, it remains elusive how SERT genotype modulates LFPs in reward-based decision-making. However, besides its effect on behavior, sparse evidence suggests that SERT knockout affects the morphology and activity of neurons in the OFC and AMY. This includes studies showing that SERT^−/−^ animals have greater spine density on OFC neurons and BLA pyramidal neurons (Wellman et al. 2007; Nietzer et al. 2011; Sakakibara et al. 2014), and increased c-fos staining, which likely reflected enhanced neuronal activity, in the OFC and BLA (Nonkes et al. 2010).

In this study, we propose that LFP synchronization (or functional connectivity)—i.e. an interaction index—between the OFC and AMY participates in reward-based decision-making and that this synchronization is modulated by SERT genotype. To test this hypothesis, we trained SERT^−/−^ male rats and their wildtype counterparts in an auditory discrimination task in which a correct behavioral response entailed a reward when it followed a CS+ and no outcome when it followed a CS−. We aimed thereby at understanding how the appetitive or neutral/negative values of the CS, and the outcomes were represented by OFC and AMY interactions (Shabel and Janak 2009; Wassum 2022). Simultaneous recordings of LFPs were performed in the OFC and AMY of both hemispheres to analyze phase-based synchronization within and across hemispheres. Additionally, we developed a network approach to characterize the frequency-dependent organization of the functional connectivity between these regions.

## Materials and methods

### Animals

Serotonin transporter knockout (SERT^−/−^) rats (Slc6a4^1Hubr^) were generated from a Wistar background by N-ethyl-N-nitrosourea (ENU)-induced mutagenesis (Smits et al. 2006; Homberg et al. 2007) and outbred with commercially available wildtype Wistar rats (Charles River Laboratories) for at least 15 generations. Seven wildtype (SERT^+/+^) and seven SERT^−/−^ male rats were obtained from SERT^+/+^ × SERT^+/+^ breedings (four SERT^+/+^ rats), SERT^−/−^ ×SERT^−/−^ breedings (four SERT^−/−^ rats), or SERT^+/−^ × SERT^+/−^ breedings (three SERT^+/+^ and three SERT^−/−^ rats). The housing rooms of the experimental rats were temperature-controlled (21°C) with a 12:12 h reverse dark/light cycle. Until surgery, the rats were housed in pairs, which were constituted of rats of the same or opposite genotype, and individually thereafter. All experimental procedures were performed during the dark phase. Rats had access to food and water ad libitum until the start of behavioral training (weight: 350 to 450 g). They were then gradually habituated to a limited water access schedule over the course of 3 d until receiving ad libitum water access for only 15 min per day during training days (2 h after the end of the training session) and 60 min on no-training days. Rats’ body weight was thereby maintained to ~85% to 90% of their free-feeding body weight. Experimenters, blind to the genotype, handled the rats 10 to 15 min/d for 5 d before starting behavioral training in the experimental room and left them there for 1 h to habituate. All experiments were conducted in accordance with Directive 63/2010/EU and approved by the Central Committee on Animal Experiments (Centrale Commissie Dierproeven, CCD, The Hague, The Netherlands) with all efforts being made to minimize animal suffering.

### Behavioral procedures

#### Apparatus

**Fig. 1:**
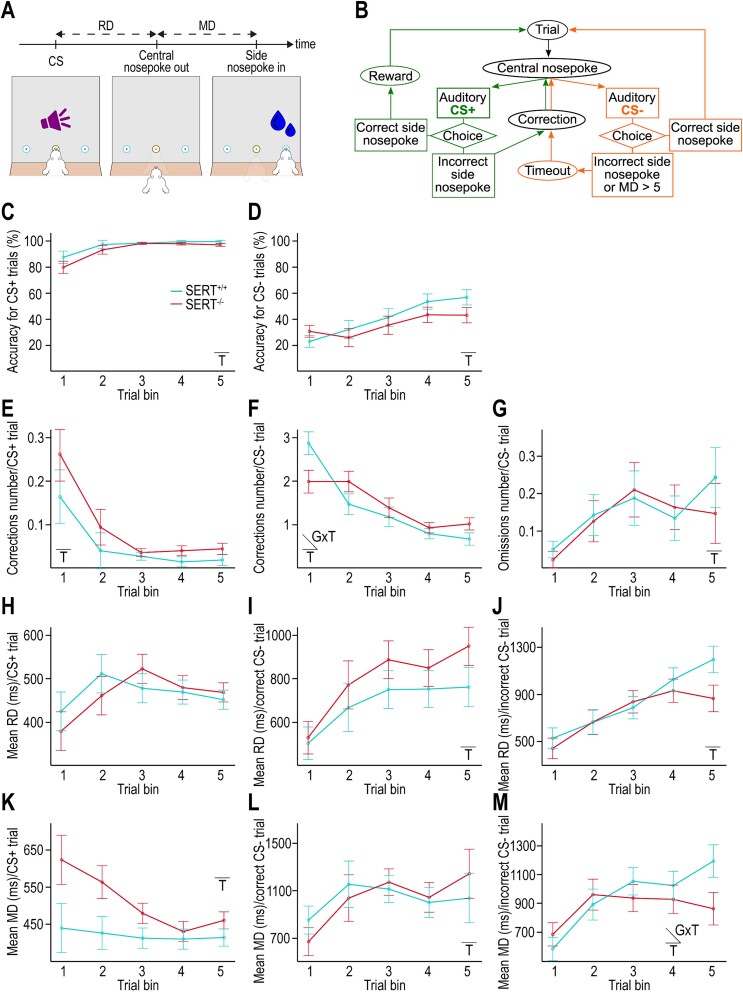
SERT genotype does not significantly influence the acquisition of the auditory discrimination task. A) Diagram of the auditory discrimination task depicting RD and MD. B) Workflow diagram of the auditory discrimination task. C, D) Accuracy for CS+ and CS− trials, respectively. Accuracy is calculated as the percentage of correct behavioral responses at first attempt (i.e. excluding corrections) over the first 5 trial bins of 100 successfully completed CS+ or CS− trials (i.e. including corrections). E, F) Number of corrections per CS+ and CS− trials, respectively, computed as the number of incorrect responses before a successful CS+ or CS− trial completion. G) Number of omissions per CS− trial computed as the average number of lack of response within 5 s following the CS− before a successful CS− trial completion. H–J) RD for CS+ trials, CS− trials with incorrect response and CS− trials with correct response, respectively. RD is calculated over the first 5 bins of 100 CS presentations for each task condition. K–M) MD for CS+ trials, CS− trials with incorrect response, and CS− trials with correct response, respectively. MD is calculated over the first 5 bins of 100 CS presentations for each task condition. C–M) Insets indicate when an effect is statistically significant (T = training, GXT = interaction genotype × training) (*n* = 7 SERT^+/+^ rats, *n* = 7 SERT^−/−^ rats).

Figure 1A shows a graphical representation of the apparatus. Rats were trained in conditioning operant chambers with attenuation of external light and noise. One wall of the chamber was equipped with three horizontally positioned ports—each containing a green LED and a near-infrared beam to detect nose-poke entries, two green LEDs, and two speakers situated above the left- and right-side ports. The side ports were connected to a pump delivering ~50 μl of water when activated. A camera mounted on the chamber ceiling enabled the experimenters to monitor the rats during task execution. The experimental room was kept dark except for a red light to maintain experimenter visibility. All steps of the task were programmed and run in MATLAB 2019b (The Mathworks Inc., Natick, MA, USA) using an in-house code.

#### Initial training of operant conditioning

Rats were progressively habituated to stay in the operant chamber for 1 h (in daily 15 min increments) and then daily trained five times per week on most weeks. First, rats learnt to initialize trials by introducing their nose in the central port for a minimum of 500 ms (in 100 ms increments) and to obtain a reward (~50 μl of water) by subsequently introducing their nose in the randomly assigned correct side port (behavioral response). The central LED was lit at the beginning of each session until a successful trial initialization occurred and then after each trial completion until the next successful initialization. Each session consisted of up to 100 successfully completed trials randomly drawn from two sets of 50 trials with water release in one of the two side ports or terminated after a maximum of 1 h. At this stage, the rats could freely test each port until receiving the reward. The completion criterion of this first pretraining phase was that rats executed at least 90 successful trials (i.e. 90 earned rewards) in each of three consecutive sessions.

The second pretraining phase varied in three ways from the first pretraining phase: (i) Only a maximum of four trials could be consecutively rewarded on the same side to prevent side preference, (ii) successful trial initializations were accompanied by a “neutral” sound (5 kHz, 1 s, 70 dB) to habituate the rats to sounds in the chamber, and (iii) correction trials were introduced. Specifically, following an incorrect response, rats had to repeat the sequence of initialization and side nose-poke until the correct response was made. An absence of any side nose-poke within 5 s of trial initialization was also deemed an incorrect response and followed by a 5 s timeout during which all five LEDs were lit, white noise was played, and no new trial initialization was made possible. The completion criterion for this second pretraining phase was three sessions with at least 90 successful trials (i.e. 90 earned rewards) within 1 h or less.

#### Auditory discrimination task

Figure 1B shows a flowchart of the auditory discrimination task. At each trial initialization, the rats had to discriminate between two distinct auditory conditioned stimuli (CS: 2 or 8 kHz, 1 s, 70 dB) and to respond by selecting a nose-poke side port (choice). The CS+ was associated with the reward (water) when the correct side port was chosen by the rat. The CS− was associated with no reward when the correct (alternative) side port was chosen by the rat. In both cases, choosing the incorrect side ports led to no reward. The CS+ and CS− tones and their respective correct nose-poke ports were predefined per rat and counterbalanced within genotypes. The 1 h sessions started with two trials with CS+ presentations and continued with CS presentations in a pseudorandom order with a maximum of four consecutive trials with the same CS and a correct response. Rats could do more than 100 successfully completed trials per session (which was not equivalent to 100 rewards as CS− successful completion involved no reward) if this number was achieved in less than 1 h. Correction trials followed incorrect responses for both CS+ and CS− trials. The absence of a behavioral response within 5 s after CS− trial initialization was deemed incorrect and recorded as an omission. After all CS− incorrect trials, a 5 s timeout with all LEDs lit and white noise came was implemented to encourage correct responses. The completion criterion of the auditory discrimination task was a minimum of three sessions with an average accuracy score equal to or higher than 60% for CS− trials (the accuracy score excluded correction trials by definition), as the accuracy score for CS+ trials was higher for all animals. Next, the rats underwent surgery for LFP electrodes implantation (described below). Following surgery and recovery, the rats performed the same auditory discrimination task while LFPs were recorded with no criterion expected to be met.

### Behavioral data analysis

For all sessions of the discrimination task (pre- and postsurgery), the sequence of CS presentations and behavioral response sides, reaction duration (RD, i.e. duration between trial initialization/CS onset and release of the central port), movement duration (MD, i.e. duration between release of the central port and behavioral response) (Fig. 1A), intertrial intervals (ITIs), and the number of corrections and omissions were continuously collected. Following brain surgery, together with LFP data acquisition, we also stored the number of anticipatory trial initializations (i.e. nose-pokes in the central port during timeouts) and failed trial initializations (i.e. central nose-pokes < 500 ms during ITI).

**Fig. 2:**
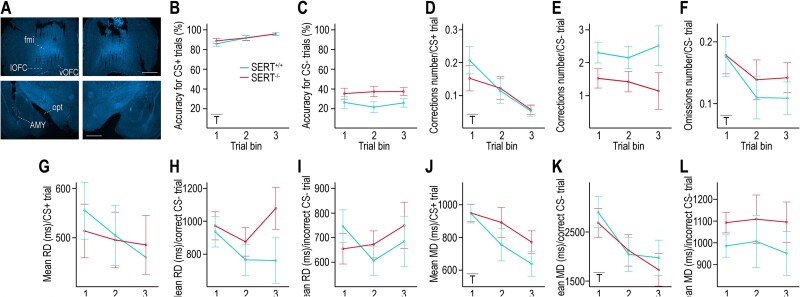
Behavioral parameters during performance of the auditory discrimination task with simultaneous LFP recordings. A) Representative images of DAPI-stained slices of each hemisphere (left and right) containing the OFC or the AMY (top and bottom, respectively) from a SERT^−/−^ rat’s brain with visible electrode tracks. B, C) Accuracy for CS+ and CS− trials, respectively, calculated as the percentage of correct behavioral responses at first attempt (i.e. excluding corrections) over 300 successfully completed CS+ or CS− trials (i.e. including corrections). A trend for higher accuracy during CS− trials is seen in SERT^−/−^ rats. D, E) Number of corrections per CS+ and CS− trials, respectively. A trend for lower number of corrections during CS− trials is seen in SERT^−/−^ rats. F) Number of omissions (lack of response within 5 s) per CS− trial. G–I) RD for CS+ trials, CS− trials with incorrect response, and CS− trials with correct response, respectively. J–L) MD for CS+ trials, CS− trials with incorrect response, and CS− trials with correct response, respectively. RD and MD are calculated over the first 3 bins of 100 CS presentations for each task condition. A) Scale bar (in white) = 1 cm. B–L) Insets indicate when an effect is statistically significant (T = training) (*n* = 6 SERT^+/+^ rats, *n* = 7 SERT^−/−^ rats).

The behavioral dataset was processed with MATLAB 2019b. To detect a training effect on performance during the presurgery phase, the mean of each behavioral measure was computed over the first five bins of 100 successfully completed trials with each CS (CS+ or CS−), unless otherwise specified. This value was close to the common minimum number of collected data for all animals in the presurgery phase (before reaching the criterion for surgery) (Fig. 1). Following surgery, the means were calculated over the first three bins of 100 successfully completed trials with each CS, as the minimum number collected in every rat (Fig. 2). When the rats made more than one incorrect response before successfully completing a trial, we first averaged the behavioral measures over these incorrect responses. The accuracy score per CS was defined as the percentage of correct responses at first attempt. The mean number of omissions was only calculated for CS− trials, as omissions in case of CS+ trials were very rare. RD and MD were sorted according to CS+ trials, CS−−trials with correct response, and CS− trials with incorrect response. MD was excluded from analysis when exceeding 10 s after CS+ onset (~1–5 values/rat) and 5 s after CS− onset. To compute ITIs, we only used the values for trials that were not corrections (but could have either correct or incorrect responses) and sorted them accordingly to the CS of the preceding trial. The first 500 and 300 trials for each (preceding) CS were averaged in the pre- and postsurgery phases, respectively. During postsurgery, we averaged the number of failed trial initializations over the first 300 completed trials of any CS and the number of anticipatory trial initializations over the first 300 completed CS− trials (no timeout for CS+ trials).

### Electrodes manufacture

#### Design

We adapted the method by França et al. to manufacture wire electrode arrays targeting either the orbitofrontal cortex (OFC) or the basolateral amygdala (BLA) in both hemispheres simultaneously (Fig. S1A) (França et al. 2020). Due to the small size of the BLA and the possible integration of LFP signal from up to several hundred micrometers of brain tissue for each electrode (Xing et al. 2009), we cannot ascertain that recorded LFPs exclusively came from this structure and not also from neighboring amygdala nuclei. Therefore, we refer to the target region as amygdala (AMY). We designed the arrays for OFC cross-hemispheric recordings as a grid of 2 rows of electrodes in the anteroposterior axis and 8 columns of electrodes in the mediolateral axis per hemisphere (total of 32 electrodes) with 2 mm separating the most medial columns of each hemisphere (Fig. S1A). The array optimized for cross-hemispheric amygdala recordings had a 5 rows × 3 columns grid design per hemisphere (total of 30 electrodes) separated by 9.1 mm between the most medial columns of each hemisphere (Fig. S1A).

#### Assembly

We inserted 50 μm-diameter insulated tungsten 99.95% wires (California Fine Wire Company) through 80 μm-diameter holes drilled 250 μm apart into three PCB stencils (Eurocircuits) that were aligned at ~1/1.5 cm from each other. The electrode wire ends (lower tips), which were not insulated and therefore able to detect LFPs, were aligned and adjusted in length with a caliper according to the desired dorsoventral (DV) coordinates of each target area (Fig. S1A). We then glued together each hemisphere’s set of electrodes using photo-activated glue (OptiBond, Kerr, Kloten, Switzerland) between the upper two alignment stencils (with respect to the electrode wire ends) and cut the upper tips to retrieve them from the top stencil. We glued the two sets of electrodes perpendicularly to the same PCB (Eurocircuits, identical design as in França et al. 2020) while maintaining them parallel and at the correct distance using the two bottom stencils. The PCB was presoldered to a 32-channels connector (A79026-001, Omnetics Connector Corporation). To finalize the implant, we slid the remaining alignment stencils off the wires, removed the wire insulation for ~1 mm from the wire ends at the PCB side and connected each wire to a different PCB through-hole using silver paint. We directly soldered the ground wire (51 μm stainless steel coated with perfluoroalkoxy alkane, Science Products, Hofheim, Germany) to the PCB. Epoxy glue was applied to protect the connections between wires and PCB and PCB and connector (Fig. S1A).

### Surgery

The rats, individually housed 3 to 5 d prior to surgery, received the steroidal anti-inflammatory drug carprofen in their drinking water (10 mg/300 ml, Rimadyl) ad libitum during 48 h before surgery. A local anesthetics cream (lidocaine/prilocaine 5%, Teva Nederland B.V., Haarlem, The Netherlands) was applied in both ears before anesthesia induction to prevent ear bar discomfort. Anesthesia induction was achieved with 5% isoflurane in a mixture of oxygen (0.25 L/min) and air (0.25 L/min) for 5 min and anesthesia maintenance with 2% isoflurane in the same mixture of oxygen and air. After shaving the head, the rats were placed in a stereotaxic frame (Kopf Instruments, Los Angeles, CA, USA). Betadine was applied to disinfect the skin. We subcutaneously injected 0.25 ml of a local anesthetics’ mixture (Lidocaine HCl, 10 mg/ml, 0.4 ml; Bupivacaine Actavis, 5 mg/ml, 0.2 ml; diluted with 3.4 ml NaCl) at the incision site before exposing the skull, cleaned with 3% hydrogen peroxide to facilitate the visualization of bregma and lambda. Five holes for the support screws (one anterior to the OFC craniotomies; two between the OFC and AMY craniotomies and two posterior to the AMY craniotomies) and one hole for the ground screw (over the cerebellum) were drilled and screws were inserted accordingly. Four holes at the corners of each of the four craniotomies (OFC and AMY in both hemispheres) were drilled at stereotactic coordinates calculated to accommodate the electrode arrays. Thereafter, the four craniotomies were drilled using a 0.5 mm drill bit. The OFC implant was lowered first (electrodes per hemisphere centered around: AP: +3.7 mm, ML: +/−1.9 relative to bregma, DV: −4.4 mm relative to brain surface) (Fig. S1A). We protected the brain surface with a drop of Vaseline before applying dental cement to maintain the implant and screws in place (first layer: Super-Bond C&B, Sun Medical, Moriyama, Japan, second layer: Paladur, Kulzer GmbH, Hanau, Germany). We repeated the procedure for the AMY implant (electrodes per hemisphere centered around: AP: −2.6 mm, ML: +/−4.8 mm, DV: −7.9 mm relative to brain surface) (Fig. S1A). Rats received 2 ml subcutaneous saline injections every hour throughout the procedure, and a subcutaneous carprofen injection (10 mg/kg) 30 min before the end of surgery, repeated on days 1 and 2 postsurgery (~22 h after the previous injection). Rats’ weight and visual appearance were carefully monitored for 3 d postsurgery. One of the SERT^+/+^ rats did not recover from surgery. Therefore, six SERT^+/+^ rats and seven SERT^−/−^ rats were included in the rest of the experimental procedures, data acquisition, and analysis.

### Electrophysiology data acquisition and preprocessing

At the start of each session, rats were gently restrained using a towel to connect each implant connector to an RHD 32-channel recording headstage attached to an RHD dual headstage adapter (Intan Technologies, Los Angeles, CA, USA). A commutator (Adafruit, New York, NY, USA) maximizing the animals’ freedom of movements in the operant chamber was soldered to an RHD SPI interface cable (Intan Technologies, Los Angeles, CA, USA) passing through the ceiling of the box and connecting the headstage adapter to an Open Ephys acquisition board (Siegle et al. 2017). Electrophysiology data were acquired at a sampling rate of 30 kHz. Time markers, i.e. central port nose-poking initiation, CS onset (500 ms later), reaction time (RT, i.e. time point from CS onset marking the release of the central port), and movement time (MT, i.e. time point from CS onset marking the behavioral response), synchronized electrophysiological data with behavior.

We preprocessed and analyzed all LFP data offline using MATLAB 2019b. LFP data were down-sampled to 1 kHz, high-pass-filtered at 0.5 Hz and notch-filtered at 50 and 100 Hz to reduce line noise. Epochs encompassing −2 to 5 s around CS onset were extracted. The average reference computed per brain area and hemisphere was subtracted from each electrode in the corresponding area. We used EEGLAB (Delorme and Makeig 2004) to visually inspect the data and exclude artifactual epochs (due, for example, to movement artifacts) and excessively noisy channels. Across all sessions for all animals, the median rate of epoch rejection was 16% and the median number of rejected channels was 4. We ran independent components analysis (ICA) on the remaining data to remove repetitive and multichannel artifactual components such as licking artifacts (median number of removed components = 6). Finally, we excluded trials (with a median rate of 12.4%) with an anticipatory RT (<100 ms) or with an RT or MT above 3 SD from the mean.

### Electrophysiological data analysis

Time–frequency analysis was applied on the cleaned, epoched, and average-referenced LFP data using temporally narrowband filtering via convolution with complex Morlet wavelets. These wavelets were defined as time-domain Gaussians with a number of cycles varying logarithmically from 6 to 20 in 50 steps with increasing frequencies. Extracted frequencies ranged from 2 to 80 Hz in 50 logarithmically spaced steps. To investigate task-related changes in neural activity, data were time-locked to CS onset, RT, or MT. To discern condition-specific neural activity, we separately analyzed the three task conditions, i.e. CS+ trials with correct response, CS− trials with correct response, or CS− trials with incorrect response. The average number of trials per animal for the three conditions was: 506 ± 115, 440 ± 123 and 536 ± 75 for SERT^+/+^ rats and 409 ± 106, 393 ± 114 and 279 ± 69 for SERT^−/−^ rats, respectively. We focused our LFP analysis on phase synchronization, as synchronization of neural oscillations’ phases is hypothesized to sustain efficient exchange of information across brain areas (Fries 2005). Specifically, we computed intersite phase clustering (ISPC), a measure of functional connectivity between brain regions relying on trial-average phase angle differences between pairs of electrodes, at each time point (Cohen 2014). For the three different time-locks of the data, ISPC values were calculated in steps of 20 ms (from −1,200 to +1,500 ms around CS onset, from −1,000 to +1,000 ms around RT, or from −1,000 to +500 ms around MT).

#### Time–frequency ISPC analysis

**Fig. 3:**
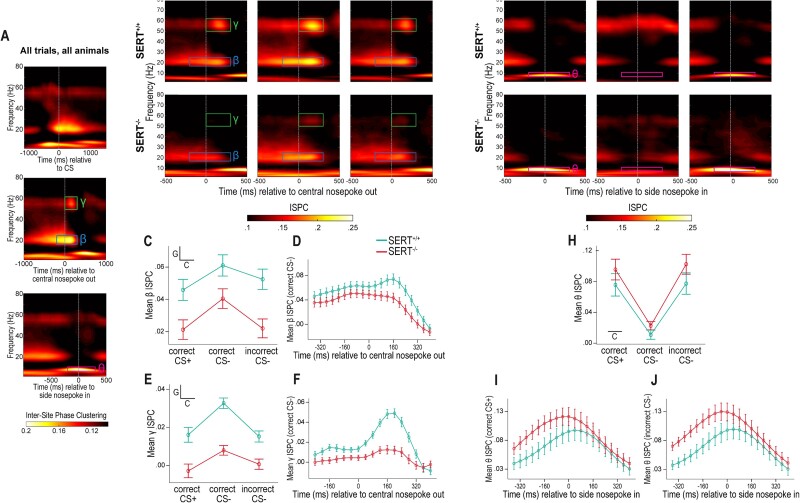
SERT^−/−^ genotype reduces task-related changes in beta and gamma inter-site phase clustering between the OFC and AMY. A) Visual detection of task-related changes in intersite phase clustering independently of task condition and genotype. Time–frequency representations of ISPC changes relative to the baseline period (−1,100 to −700 ms relative to CS onset) between OFC-AMY electrode pairs averaged over all task conditions and animals from both genotypes. Representations are time-locked to CS onset (top), RT (middle), or MT (bottom). Boxes delineate time-frequency windows of interest in this article, i.e. of increased beta, gamma, and theta synchronization, respectively. B, G) Time–frequency representations of the same ISPC changes as in Fig. 3A when time series are locked to RT and MT (middle and bottom, respectively) but averaged over trials within a task condition and over animals within a genotype. The upper and lower rows are for SERT^+/+^ and SERT^−/−^ rats, respectively; the left, central and right columns are for trials with CS+ and correct response, CS− and correct response, and CS− and incorrect response, respectively. C, E) Profile plots of the ISPC averages over time and frequency within the delineated gamma and beta boxes of Fig. 2B, respectively. SERT^+/+^ rats have higher OFC-AMY beta and gamma connectivity for all three task conditions than SERT^−/−^ rats. Trials with CS− and correct response elicit more intense beta and gamma connectivity. D) Profile of the ISPC changes in beta connectivity from −360 to +400 ms around RT averaged over correct CS− trials and over animals within genotype. The timepoint of maximal beta connectivity occurs later in SERT^+/+^ rats than in SERT^−/−^ rats. F) Profile of the ISPC changes in gamma connectivity from −240 to +400 ms around RT averaged over correct CS− trials and over animals within genotype. The averaged peak times are not different across genotypes. H) Profile plot of the ISPC averages over time and frequency within the delineated theta boxes of Fig. 2G. Both genotypes have strong OFC-AMY theta synchronization in case of correct CS+ and incorrect CS− trials, i.e. when rats nose-poke in the side port where water can be obtained. I, J) Profile of the ISPC changes in theta connectivity from −360 to +400 ms around MT averaged over correct CS+ trials I) or incorrect CS− trials J) and over animals within genotype. The timepoints of maximal theta connectivity are similar in SERT^+/+^ and SERT^−/−^ rats. Insets in profile plots C, E, H) indicate when an effect is statistically significant (C = task condition, G = genotype) (*n* = 6 SERT^+/+^ rats, *n* = 7 SERT^−/−^ rats).

Only the interareal pairs of electrodes between the OFC and AMY were included (in other words, electrode pairs within each brain area or between OFC of both hemispheres or AMY of both hemispheres were excluded) (Fig. 3, Fig. S2). For each rat, the ISPC values were first averaged over pairs of electrodes while applying a weight that was inversely proportional to the corresponding number of missing trials (stored during preprocessing). We then created two sets of ISPC values, one by averaging over all trials (set 1) and the other by averaging over trials of the same condition (set 2). We next averaged the time–frequency (TF) ISPC values of set 1 over all animals (Fig. 3A) and of set 2 over all animals/genotype (Fig. 3, Fig. S2). For comparisons across conditions and genotypes, frequency-band and condition-specific baseline averages of ISPC values were calculated over a pre-CS onset period from −1,100 to −700 ms and subtracted from synchronization values in the corresponding frequency-band window. To draw the temporal profiles, we averaged ISPC values over two consecutive time points (every 40 ms) (Fig. 3).

#### Functional network analysis

**Fig. 4:**
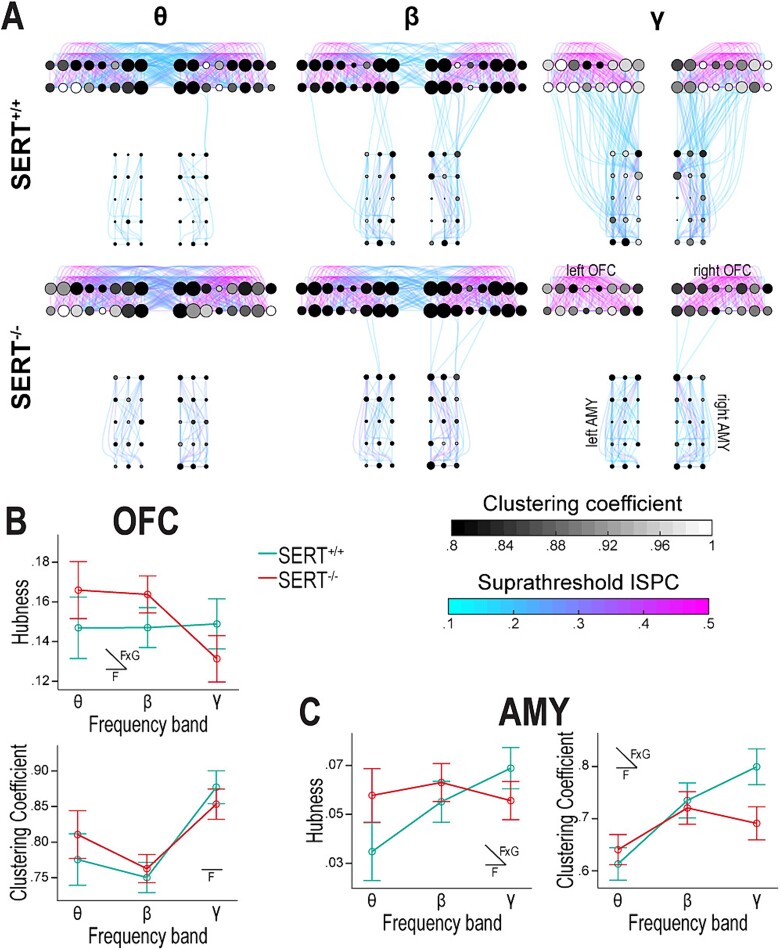
During the baseline period, the organization of the cross-hemispheric OFC-AMY functional network is frequency-dependent and SERT^−/−^ rats display a weakly connected network in gamma. A) The 6 graphs represent the cross-hemispheric OFC-AMY network with each node depicting an electrode (8 columns × 2 rows in each OFC, 3 columns × 5 rows in each AMY). The solid curves represent the suprathreshold connectivity between electrode pairs. The node radius is proportional to hubness, and the fill color, to clustering coefficient. The upper row is for SERT^+/+^ rats (*n* = 6) and the lower one for SERT^−/−^ rats (*n* = 7); the left, central, and right columns are for the theta (7 to 11 Hz), beta (17 to 25 Hz), and gamma (50 to 62 Hz) frequency bands corresponding to task-related increases in synchronization. The functional network organization shows less cross-hemispheric OFC and more ipsilateral OFC-AMY interactions with increasing frequencies during the baseline period, particularly in SERT^+/+^ rats. SERT^−/−^ rats have very scarce ipsilateral OFC-AMY gamma connectivity. B, C) Profile plots of hubness and clustering coefficient averaged over brain regions (combining both hemispheres) and over animals within genotype. Both properties vary with the frequency bands in the OFC B) and AMY C). These variations are also dependent on the genotype for hubness in both brain regions and clustering coefficient in the AMY. The latter is decreased in SERT^−/−^ compared to SERT^+/+^ rats in gamma C, right). Insets in profile plots indicate when an effect is statistically significant (F = frequency, FxG = interaction frequency × genotype) (*n* = 6 SERT^+/+^ rats, *n* = 7 SERT^−/−^ rats).

**Fig. 5:**
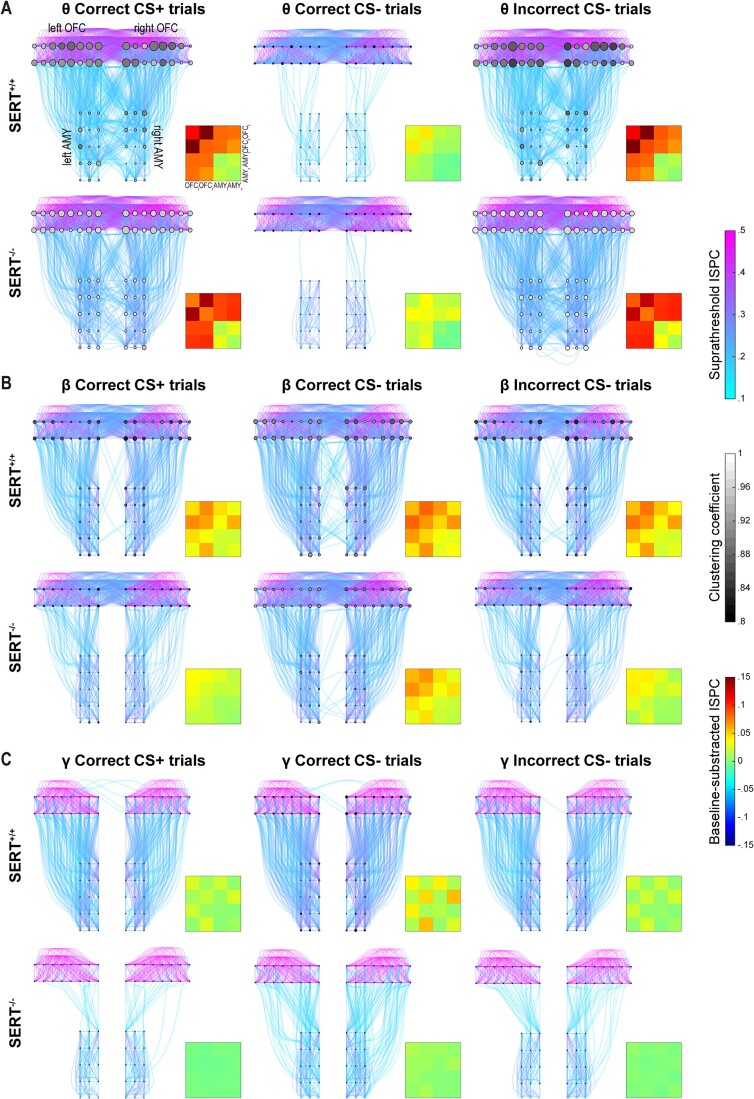
SERT knockout reduces task-related functional connectivity dynamics in beta and gamma within the cross-hemispheric OFC-AMY network while preserving theta dynamics. The 18 graphs represent the cross-hemispheric OFC-AMY network with the same elements as in Fig. 4. 4-by-4 matrices of suprathreshold baseline-subtracted ISPC values are shown in bottom-right insets. Each colored square (jet color map) shows the average over all pairs of electrodes between two brain regions (OFC_l_ = left OFC, OFC_r_ = right OFC, AMY_l_ = left AMY, AMY_r_ = right AMY). A–C) The upper and lower row is for SERT^+/+^ (*n* = 6) and SERT^−/−^ (*n* = 7) rats, respectively; the left, central and right columns are for trials with CS+ and correct response, CS− and correct response, and CS− and incorrect response, respectively. A) Theta connectivity around MT is influenced by task condition but not by genotype, with responses to the side port associated with rewards (left and right columns) showing much richer network connectivity compared to responses to the side port associated with absence of rewards (central column) in both genotypes. B) Beta functional network connectivity around RT, predominantly cross-hemispheric between the OFCs and ipsilateral between OFC and AMY, is more intense in SERT^+/+^ rats than in SERT^−/−^ rats and reinforced when the trial’s correct completion involves no reward (central column) compared to the other task conditions. C) Gamma functional network connectivity around RT is mainly ipsilateral between OFC and AMY and shows similar genotype and task condition effects as in Fig. 5B.

We first generated “all-to-all” connectivity matrices based on the ISPC values averaged within time- and frequency-windows and over trials between each pair of electrodes (Figs. 4 and 5, Fig. S3). We then thresholded these matrices by keeping all synchronization strengths above the median and reiterated this thresholding when averaging over animals. The resulting suprathreshold matrices were converted into network graphs. We calculated “hubness” for each electrode as the number of suprathreshold connections connecting one electrode to the network divided by the total number of possible connections (i.e. 61, of which the mean number of rejected channels during preprocessing for each animal was subtracted). To calculate the clustering coefficient for each electrode, the first step was to identify the subset of electrodes that had suprathreshold connectivity to that electrode. In a second step, we counted the number of suprathreshold connections within this subset of connecting electrodes and divided this number by the total number of connections that could possibly exist between them (Cohen 2014).

### Histology

At the end of the experiment, the rats were briefly anesthetized with 5% isoflurane before receiving an intraperitoneal injection of pentobarbital and transcardially perfused with 0.01 M phosphate-buffered saline (PBS) followed by 4% paraformaldehyde (PFA). Brains were dissected, and the electrode implants were extracted before or after being postfixed in 4% PFA. Sixty-micrometer brain sections were done using a Leica VY100S vibratome (Leica Biosystems, Wetzlar, Germany). Free-floating sections containing the OFC, AMY, or visible electrode tracks were stored in 0.01 M PBS, stained with 4′,6-diamidino-2-phenylindole (DAPI) (Thermo Fisher Scientific, Waltham, MA, USA) and mounted on slides with a mounting medium (Merck Life Science N.V., Amsterdam, The Netherlands). Images of the electrode target sites were acquired using a Zeiss Axio Imager 2 microscope with a 2.5× objective, charge-coupled device (CCD) camera, and ZEN 3.2 microscopy software. We confirmed that all animals included in the analysis had electrodes situated in the vicinity of the OFC and AMY of both hemispheres (Fig. 2A and Fig. S1B and C).

### Statistical analysis

The statistical analysis was primarily aimed at comparing data across genotypes. Behavioral data were statistically analyzed using IBM SPSS by two-tailed Mann–Whitney U tests or mixed-model ANOVAs with genotype as between-subject factor and bin of trials, CS type, condition, or frequency as within-subject factors, as appropriate. The threshold chosen for statistical significance was a *P*-value ≤ 0.05. Before ANOVAs, all conditions were tested for the absence of outliers, normality of distribution (Shapiro–Wilk test), and equality of variances (Levene’s test). In mixed-model ANOVAs, the Greenhouse–Geisser correction was used as sphericity was not assumed. MATLAB or SPSS were used for data visualization and graphs. In the main text, values are reported as mean ± SD.

## Results

### Auditory discrimination task learning in SERT^+/+^ and SERT^−/−^ rats

We first examined whether both SERT^+/+^ and SERT^−/−^ rats were able to learn to discriminate two distinct auditory CSs (CS+ and CS−) associated with distinct behavioral responses (opposite-side nose-pokes) and distinct outcomes (reward or no reward, respectively) (Fig. 1B). In the learning phase (presurgery), we split the first 500 successfully completed trials of each CS into five equal bins to assess the effects of training and genotype on rats’ performance. Accuracy, defined as the percentage of responses for which the rats selected the correct nose-poking side without needing correction trials, was our primary measure of task acquisition. While accuracy for both CSs was significantly influenced by training, no significant genotype nor interaction effects were detected (Fig. 1C and D, Table S1). In the last bin of analyzed trials (trials # 401 to 500, for which we had data for all animals as they had not reached the completion criterion), SERT^+/+^ and SERT^−/−^ rats reached an accuracy of 98.86 ± 1.07 and 96.43 ± 3.95% in CS+ trials and 56 ± 5.39 and 42.29 ± 21.24% in CS− trials, respectively (Fig. 1C and D). Although it took more trials for SERT^−/−^ than SERT^+/+^ rats (883.43 ± 962.12 and 511.29 ± 313.59, respectively) to reach 60% accuracy in CS− trials—calculated on a moving average with a window of 100 trials—this trend was not statistically significant.

Another measure of task acquisition was the mean number of corrections, i.e. repetitions of incorrect responses for a given CS. The number of corrections decreased over bins of trials with CS+ and CS− during the learning phase, which corresponded to a significant effect of training. Genotype did not significantly influence the corrections number for either CS, but there was a significant interaction effect between training and genotype for CS− trials with an apparently steeper negative slope in SERT^+/+^ than SERT^−/−^ rats (Fig. 1E and F, Table S1). The mean number of corrections was only noticeably different between genotypes in the first bin of 100 completed CS− trials. That is, it was smaller in SERT^−/−^ rats than in SERT^+/+^ rats (Fig. 1E and F; SERT^+/+^ rats: 2.85 ± 0.87, SERT^−/−^ rats: 1.97 ± 0.46). Training had also a significant effect on omissions following CS− presentations with an inverse tendency to that of corrections (Fig. 1G; Table S1), likely reflecting an increasing lack of motivation to respond in case of a CS− trial. The effects of genotype and training × genotype interaction on omissions were both nonsignificant (Table S1). Taken together, these results suggest that although SERT^−/−^ rats were less inclined than SERT^+/+^ rats to repeat erroneous responses in trials for which no reward should be anticipated in the very early phase of task acquisition, SERT^+/+^ rats may ultimately show a trend to learn faster the correct behavioral response to the CS−. However, the overall improving performance showed no significant influence of genotype and both SERT^+/+^ and SERT^−/−^ rats acquired the auditory discrimination task accordingly to the task acquisition criterion.

We also assessed the influence of training and genotype on ITIs that were self-determined by the rats. We presumed ITIs to be longer after reward delivery because of the time the rats needed to lick the water, which was confirmed by the data (first bin of trials: average ITI across all rats = 20.39 ± 7.26 s following CS+ trials, 7.1 ± 3.25 s following CS− trials). Therefore, we analyzed ITIs separately following CS+ and CS− trials. While the genotype and the interaction training × genotype had no significant effect, ITIs following trials of either type decreased with training in SERT^+/+^ and SERT^−/−^ rats alike (last bin of trials: 1. 13.7 ± 7.88 in SERT^+/+^ rats and 16.52 ± 8.1 s in SERT^−/−^ rats following CS+ trials; 2. 2.56 ± 0.62 in SERT^+/+^ rats and 5.42 ± 5.29 s in SERT^−/−^ rats following CS− trials. Table S1), which possibly also reflected task learning.

Finally, we collected on each trial the reaction duration (RD), i.e. the duration between stimulus onset and the rat’s nose release from the central port, and the movement duration (MD), i.e. the duration between the release at the central port and the response made by nose-poking in one of the side ports (Fig. 1A). We analyzed RD and MD separately according to the types of CS and of response, i.e. according to the task conditions. An effect of training on RD and MD was observed in all task conditions, statistically significant for all but for the RD in CS+ trials (Fig. 1H–M, Table S1). The RDs in CS+ trials and in CS− trials with correct and incorrect responses showed no genotype nor interaction effect and globally increased over trial bins, paralleling accuracy (Fig. 1H–J). These results suggest that a reduction in the speed of stimulus integration and/or response selection helped the rats improve accuracy. In contrast, although the MD in CS− trials with correct and incorrect responses also increased over trial bins, an overall decrease in MD was shown in CS+ trials (Fig. 1K–M). Thus, the MD might be reflective of the rat’s evolving motivation to complete trials, higher for CS+ trials than for CS− trials as no reward was delivered. While genotype and training × genotype had an almost significant effect on the MD in CS+ trials with SERT^−/−^ rats showing slower speed of response in the initial trial bins (Fig. 1K, Table S1), the influence of training on MD was different in both genotypes for CS− trials with incorrect responses and SERT^+/+^ rats eventually showed a slower speed of response in the last trial bin (SERT^+/+^ rats: 1,189 ± 302 ms, SERT^−/−^ rats: 859 ± 294 ms).

### Auditory discrimination task performance during LFP recordings in SERT^+/+^ and SERT^−/−^ rats

Next, we studied the rats’ performance in the task after they had undergone brain surgery and while LFP recordings were acquired. The first 300 successfully completed trials of each CS were split into three bins of 100 trials. Accuracy for CS+ trials was high in both genotypes from the first trial bin (SERT^+/+^ rats: 85.17 ± 7.78%, SERT^−/−^ rats: 88.14 ± 5.73%) suggesting that the rats had accurately retained the association CS+/behavioral response/reward (Fig. 2B, Table S2). Yet, training, but not genotype nor genotype × training, had a significant effect leading to a similar and higher accuracy for CS+ trials in SERT^+/+^ and SERT^−/−^ rats in the last trial bin (95.17 ± 4.45 and 95.29 ± 2.69%, respectively). Correspondingly, the number of corrections for CS+ trials decreased with training and was similarly very low in both genotypes in the last trial bin (Fig. 2D, Table S2). In contrast, the accuracy and the number of corrections for CS− trials were not influenced by training but both showed a trend for a genotype effect that did not reach statistical significance (Fig. 2C and E, Table S2). SERT^−/−^ rats tended to be more accurate and to repeat fewer erroneous responses in the case of CS− trials than their wildtype counterparts (last trials’ bin for SERT^+/+^ and SERT^−/−^ rats, respectively: 1. accuracy: 25 ± 12.92 and 36.57 ± 8.16%; 2. number of corrections: 2.5 ± 2.18 and 1.11 ± 0.23. Figure 2C and E). Yet, both genotypes had a similar number of omissions per completed CS− trial and only training significantly impacted it (last trials’ bin: SERT^+/+^ rats: 0.11 ± 0.06, SERT^−/−^ rats: 0.14 ± 0.07. Figure 2F, Table S2). Taken together, these results suggest that SERT^−/−^ rats may slightly outperform SERT^+/+^ rats in the association CS−/behavioral response/no reward while LFPs were recorded.

Other behavioral parameters included failed and anticipatory trial initializations and ITIs. There was no main effect for the average number of failed trial initializations (last bin of trials: SERT^+/+^ rats: 0.41 ± 0.17, SERT^−/−^ rats: 0.49 ± 0.38. Table S2). Yet, the average number of anticipatory trial initializations during timeout following CS− with incorrect responses similarly increased with training in both genotypes, which might suggest that all rats became more impatient with timeout (last bin of trials: SERT^+/+^ rats: 0.41 ± 0.11, SERT^−/−^ rats: 0.48 ± 0.23. Table S2). As in the phase of task learning presurgery, ITIs following CS+ trials were longer than following CS− trials (first bin of trials: average ITI across all rats = 42.92 ± 22.81 s following CS+ trials, 17.05 ± 10.23 s following CS− trials). It is noteworthy that in both cases, ITIs were longer than before surgery. There was a tendency for and a statistically confirmed effect of training on ITIs following CS+ and CS− trials, respectively, with no effect of genotype or interaction as in presurgery (last bin of trials: after CS+ trials: SERT^+/+^ rats: 37.44 ± 29.55 s, SERT^−/−^ rats: 21.1 ± 14.04 s; after CS− trials: SERT^+/+^ rats: 6.57 ± 3.36 s, SERT^−/−^ rats: 5.13 ± 2.24 s, Table S2).

Finally, the training effect for RD and MD was collectively less pronounced during LFP recordings than during the task-learning phase, suggesting that the behavioral response globally stabilized (Fig. 2G–L, Table S2). Only MD following CS+ presentations or following CS− presentations with correct behavioral responses showed such a training effect, with MD shortening over trial bins in both genotypes (Fig. 2J and K, Table S2; (i) CS+ trials: 1st and 3rd trial bin, respectively: SERT^+/+^ rats: 943 ± 141 and 635 ± 71 ms; SERT^−/−^ rats: 946 ± 143 and 768 ± 241 ms. (ii) CS− trials with correct response: 1st and 3rd trial bin, respectively: SERT^+/+^ rats: 2,905 ± 953 and 1,976 ± 1,028 ms, SERT^−/−^ rats: 2,687 ± 584 and 1,730 ± 728 ms), contrasting the overall lengthening of RD and MD in presurgery (Fig. 1H–M). Then, the strategy of modulating MD during the postsurgery phase for these two task conditions (with very high and 100% response accuracy for CS+ and CS− trials, respectively) seemed only to be aimed at speeding responses. No significant genotype nor interaction effects were found during the LFP recordings phase. However, RD in the case of CS− trials with correct responses displayed a diverging trend between genotypes over trial bins, with SERT^−/−^ rats having longer RD than SERT^+/+^ rats in the last trial bin (SERT^+/+^ rats: 756 ± 341 ms, SERT^−/−^ rats: 1,075 ± 338 ms; Fig. 2H, Table S2). In line with the findings on accuracy and number of corrections, this may suggest that SERT^−/−^ rats favored accuracy of stimulus integration/response selection over speed specifically in trials with CS− (Fig. 2C and E).

Collectively, the data supported that SERT^+/+^ and SERT^−/−^ rats had retained the auditory discrimination task in a similar manner overall following surgery. Yet, SERT^−/−^ rats exhibited a tendency to perform slightly better in CS− trials than the wild-type (WT) counterparts.

### SERT^−/−^ rats show weaker beta and gamma functional connectivity between the OFC and AMY at the time of decision-making

After implantation surgery, we recorded LFPs in the OFC and AMY of both hemispheres during the execution of the auditory discrimination task (Fig. 2). We explored the functional connectivity between the OFC and AMY by looking at task-related variations in intersite phase clustering (ISPC)—a phase-based synchronization measure—between all interareal pairs of electrodes (Cohen 2014). We specifically sought to investigate whether the SERT genotypes and the task conditions (here defined as CS+ with correct responses only, CS− with correct responses, and CS− with incorrect responses) influenced task-related OFC-AMY functional connectivity. We temporally locked the ISPC values averaged over all task conditions and all animals of both genotypes to the CS, RT, and MT, respectively and represented them on TF plots (Fig. 3A, top, center, and bottom plot, respectively). A visual inspection of the ISPC TF plot temporally locked to the CS revealed intense synchronization between the OFC and AMY in the theta band during trial initializations (preceding the CS) and in the beta band around the time of CS onset (top plot, Fig. 3A). Because a possible effect of the task condition is mostly irrelevant before CS integration, we did not quantitatively assess the ISPC TF plots separately for each condition and genotype. Nevertheless, we qualitatively observed that theta band synchronization during trial initializations seemed more intense in SERT^−/−^ rats (bottom row) than in SERT^+/+^ rats (top row) (Fig. S2).

In the TF representation temporally locked to RT, we identified by visual inspection two narrow windows of task-related heightened synchronization between the OFC and AMY in the beta (−200 ms to +300 ms from RT, 17 to 25 Hz) and gamma (0 to 300 ms from RT, 50 to 62 Hz) frequency bands (Fig. 3A, central TF plot). RT, the time point of central port nose-poke release that immediately precedes the rats’ movement to the left or right port initiating behavioral response, was hereafter interpreted as the time point of response selection or decision-making. We then generated the TF representations of ISPC values temporally locked to RT separately for each condition and genotype (Fig. 3B) and qualitatively assessed the beta and gamma windows on each plot. We observed that beta and gamma synchronization around RT was weaker in SERT^−/−^ rats (bottom row) compared to SERT^+/+^ rats (top row). We also noted that, irrespective of the genotype, more intense functional connectivity occurred for trials with CS− associated with correct responses (center column) than for the other conditions (CS+ with correct responses and CS− with incorrect responses, left and right columns, respectively). We then quantified the changes from baseline in ISPC values within each TF window (Fig. 3C and E). The effects of genotype and condition were significant in both cases [Beta window: genotype: *F*_(1,11)_ = 10.35, *P* = 0.008, condition: *F*_(1.76,19.32)_ = 13.22, *P* < 0.001, condition × genotype: *F*_(1.76,19.32)_ = 0.99, *P* = 0.38; Gamma window: genotype: *F*_(1,11)_ = 25.31, *P* < 0.001, condition: *F*_(1.22,13.47)_ = 34.73, *P* < 0.001, condition × genotype: *F*_(1.22,13.47)_ = 3.98, *P* = 0.06]. Specifically, post-hoc pairwise comparisons indicated that, for the beta window, the difference between genotypes was significant for each of the three conditions. Moreover, the CS− trials associated with correct responses had noticeably stronger beta synchronization than the other conditions in SERT^−/−^ rats (Fig. 3C). Regarding the gamma window, the mean divergence between genotypes was also significant for all conditions, and gamma connectivity was richer in CS− trials associated with correct responses than in the other conditions in both genotypes (Fig. 3E).

We next explored whether the temporal dynamics of connectivity changes from baseline were similar between genotypes in the beta and gamma windows around RT. We analyzed the task condition that displayed strongest interaction, i.e. CS− trials with correct responses, and generated the temporal profiles by only averaging over the range of frequencies within a window (and not over the range of time points). The temporal profiles confirmed weaker beta and gamma connectivity in SERT^−/−^ rats (Fig. 3D and F) and the respective peaks appeared slightly shifted toward the left in SERT^−/−^ rats, particularly in the beta window. Indeed, the mean peak times’ difference between SERT^−/−^ and SERT^+/+^ rats was only statistically significant for the beta window (Beta: SERT^+/+^ rats: 103.33 ± 39.81 ms, SERT^−/−^ rats: −31.43 ± 45.95 ms, *P* = 0.037; gamma: SERT^+/+^ rats: 176.67 ± 14.98 ms, SERT^−/−^ rats: 148.57 ± 16.25 ms, *P* = 0.21). This suggests that the peak time of OFC-AMY beta synchronization occurred prematurely in SERT^−/−^ rats.

On the whole, our results indicate that, in our auditory discrimination task, the SERT^−/−^ rats had weaker beta and gamma interactions between the OFC and AMY occurring around the time point of decision-making compared to their wildtype counterparts.

### Theta functional connectivity between the OFC and AMY in SERT^+/+^ and SERT^−/−^ rats at the time of behavioral response

Next, in the TF plot of ISPC values of all conditions and animals and temporally locked to MT, we visually identified an increase in connectivity between the OFC and AMY in the theta band (−200 to +300 ms from MT, 7 to 11 Hz) (Fig. 3A, bottom TF plot). Qualitatively, the ISPC TF plots split by genotypes and conditions showed a clear increase in theta communication between the OFC and AMY around MT in SERT^+/+^ and SERT^−/−^ rats for the CS+ trials associated with correct responses (left column) and for the CS− trials with incorrect responses (right column) but nearly absent in the case of CS− trials with correct responses (central column) (Fig. 3G). Statistically, the differences in theta connectivity changes from baseline were dependent on the task condition but not on the genotype. There was also no significant interaction effect detected between genotype and condition [Genotype: *F*_(1,11)_ = 1.96, *P* = 0.19, condition: *F*_(1.73,19.02)_ = 50.873, *P* < 0.001, condition × genotype: *F*_(1.73,19.02)_ = 0.35, *P* = 0.68] (Fig. 3H). The task conditions “CS+ trials associated with correct responses” and “CS− trials with incorrect responses” showed significantly augmented theta connectivity compared with “CS− trials with correct responses” according to Bonferroni-adjusted post-hoc comparisons (Fig. 3H). In the two conditions with a strong theta synchronization around MT, the rats expressed the same behavioral response, i.e. nose-poking in the same side port (opposite to the side port of CS− trials with correct responses). This was where a reward could be delivered, when appropriate, a few hundreds of milliseconds later. This rich theta functional connectivity between the OFC and AMY might thus be a marker of reward anticipation, regardless of whether the anticipation is correct or not.

Similarly to the analysis done with the beta and gamma windows around RT (Fig. 3D and F), we analyzed the temporal profiles of theta synchronization for the conditions showing strongest changes around MT, i.e. CS+ trials with correct responses and CS− trials with incorrect responses (Fig. 3I and J). Although a tendency of anticipatory peak times could also be observed in SERT^−/−^ rats for theta synchronization, it did not reach statistical significance (CS+ trials with correct responses: SERT^+/+^ rats: 30 ± 38.56, SERT^−/−^ rats: −42.86 ± 45.66 ms, *P* = 0.47; CS− trials with incorrect responses: SERT^+/+^ rats: 33.33 ± 33.33, SERT^−/−^ rats: −20 ± 16.9, *P* = 0.15).

Overall, the increased theta communication between the OFC and AMY around MT was only present when the behavioral response was made in the side port associated with reward delivery, and of similar intensity in SERT^+/+^ and SERT^−/−^ rats.

### Baseline functional organization of the cross-hemispheric OFC-AMY network across frequency bands and genotypes

Next, we leveraged our high-density, multi-site, and cross-hemispheric LFP recordings to perform a network analysis of the functional interaction between the OFC and AMY. Differing from our previous analysis focusing on interarial pairs of electrodes (Fig. 3, Fig. S2), we now used ISPC values between each pair of electrodes to generate “all-to-all” connectivity matrices, which were then thresholded and converted into graphs for a schematic visual representation (Fig. S3). In those graphs, the dots (nodes) depict electrodes, and the color-scaled lines (edges) depict the strength of the suprathreshold functional connectivity (Figs. 4 and 5). We also display two graph-theoretic properties per node: size-scaled hubness, which measures the degree to which the node is connected to the rest of the network, and gray-scaled clustering coefficient, a measure of the degree to which all the nodes connecting to one node are themselves interconnected, thereby forming a cluster (Figs. 4 and 5).

We first mapped the network’s functional organization during the baseline time window, i.e. shortly before trial initialization (−1,100 to −700 ms relative to CS onset), focusing our analysis on the frequency bands previously thoroughly studied: theta (7 to 11 Hz), beta (17 to 25 Hz), and gamma (50 to 62 Hz) (Fig. 4). Visible differences in interareal baseline connectivity were seen across these frequency bands in the graphs of SERT^+/+^ rats (Fig. 4A, top row). Specifically, in theta, cross-hemispheric connectivity between the OFCs was predominant; in beta, cross-hemispheric connectivity between the OFCs and ipsilateral connectivity between OFC and AMY were present; in gamma, there was only ipsilateral connectivity between OFC and AMY (Fig. 4A, top row). Moreover, intra-areal connectivity seemed substantially more prominent in the OFC than in the AMY (Fig. 4A). The interareal functional interactions during the baseline time window roughly followed the same frequency-dependent organization in SERT^−/−^ rats, except in gamma for which the ipsilateral OFC-AMY connectivity appeared nearly absent (Fig. 4A, bottom row).

We then used hubness and clustering coefficient as quantifiable indicators of network organization and averaged the corresponding measures of the nodes in the AMYs of both hemispheres and in the OFCs of both hemispheres. Statistically, the frequency band had a significant effect on both graph properties in both regions during baseline, while the genotype had no significant impact (Table S3). A significant interaction between frequency band and genotype was found for hubness in both regions and clustering coefficient in the AMY (Table S3). Hubness and clustering coefficient were either equal or superior in the theta and beta bands but inferior in the gamma band in SERT^−/−^ compared to SERT^+/+^ rats, indicating that the frequency-dependent changes in graph properties were affected by SERT genotype (Fig. 4B and C). Subsequent pairwise comparisons revealed that the AMY clustering coefficient in the gamma band was reduced during the baseline time window in SERT^−/−^ rats compared with their wildtype counterparts (SERT^+/+^ rats: 0.80 ± 0.03, SERT^−/−^ rats: 0.69 ± 0.03, *P* = 0.041). These baseline differences in gamma between genotypes were also consistent with the qualitative observation of the TF representation of ISPC values between OFC and AMY (Fig. S2).

### Functional organization of the cross-hemispheric OFC-AMY network across task conditions and genotypes during task-relevant synchronization

We next used the same network approach to study how the organization of the cross-hemispheric OFC-AMY functional network was affected by SERT genotype and the task conditions during task-related synchronization. First, we inspected the theta band synchronization window at the time of behavioral response (Fig. 3A and G). For trials with CS+ and correct response and trials with CS− and incorrect response, the functional connectivity between the two OFCs, and ipsilaterally and contralaterally between OFCs and AMYs was clearly more abundant than in trials with CS− and correct response (Fig. 5A). In the latter, the network seemed close to its baseline state (Fig. 4A), with limited interaction between OFCs and AMYs. Additionally, to facilitate the perception of changes from baseline, we condensed the network information into 4-by-4 matrices, in which the baseline-subtracted and suprathreshold ISPC values between all pairs of electrodes within one or between two areas (left and right OFCs, left and right AMYs) were averaged (Fig. 5A, inset matrices). In both genotypes, theta synchronization increased from baseline within each OFC, between OFCs, and between OFCs and AMYs (ipsi- and contralaterally) when the animals made the behavioral response in the side port associated with possible reward delivery, and showed very little change from baseline in case of correct CS− trials, when the behavioral response was in the side port never associated with reward.

Second, we inspected the windows of beta and gamma band synchronization at the time of decision-making (Fig. 3A and B). For both frequency bands, the functional organization was visually different across conditions with a denser suprathreshold network in case of trials with CS− and correct response (Fig. 5B and C). The network’s functional organization in beta, irrespective of the condition, was dominated by intra- and cross-hemispheric OFC and ipsilateral OFC-AMY connectivity. In the case of correct CS− trials, this connectivity was strengthened and supplemented with sporadic contralateral OFC-AMY connectivity in SERT^+/+^ rats (Fig. 5B). In the gamma range, the graphs showed close to absent cross-hemispheric supra-threshold connectivity, as seen during the baseline window (Fig. 4A). Most connectivity in the gamma frequency band was contained within each OFC and ipsilaterally between OFCs and AMYs, and overall, the strongest connectivity was observed during correct CS− trials in SERT^+/+^ rats (Fig. 5C). SERT^−/−^ rats showed weaker and sparser interactions in all conditions in the beta and gamma bands compared to SERT^+/+^ rats. Particularly detectable in the 4-by-4 matrices, the differences between conditions in the changes from baseline persisted to some extent in SERT^−/−^ rats in beta but were almost absent in gamma (Fig. 5B and C, inset matrices). Interestingly, the 4-by-4 matrices also revealed that the intra-areal and cross-hemispheric AMY functional connectivity was mostly unchanged compared to baseline in all conditions, frequency bands, and genotypes (Fig. 5, inset matrices).

Altogether, the cross-hemispheric OFC-AMY functional network was dynamically reorganized across task conditions. In addition, organizational differences affecting beta and gamma connectivity were visually evident between SERT^+/+^ and SERT^−/−^ rats.

### AMY and OFC of SERT^−/−^ rats display decreases in hubness and clustering coefficient in the beta and gamma bands at the time of decision-making

**Fig. 6:**
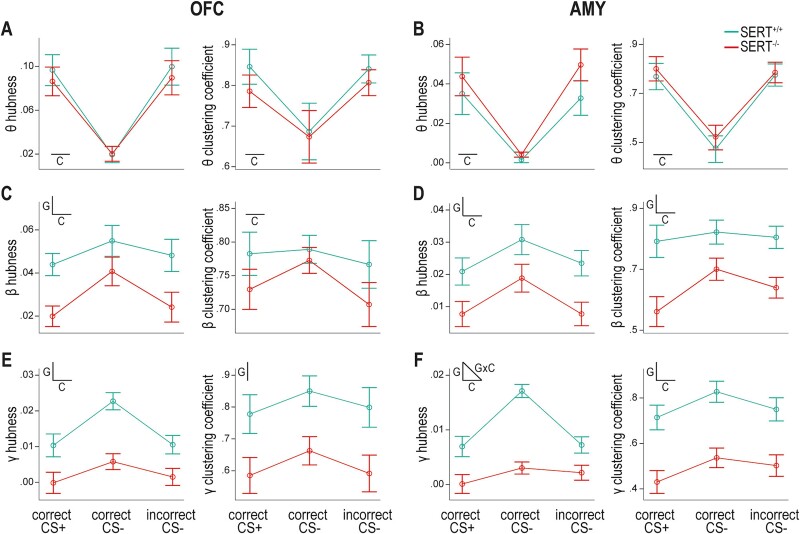
SERT genotype influences hubness and clustering coefficient during task-related increases in beta and gamma functional connectivity within the cross-hemispheric OFC-AMY network. A) Theta connectivity around the time of behavioral response is characterized in the OFC by higher hubness (left) and clustering coefficient (right) when the response is made in the side port associated with a potential reward (i.e. in trials with CS+ and correct response or CS− and incorrect response) than in the alternative side port (i.e. in trials with CS− and correct response). SERT^+/+^ and SERT^−/−^ rats show comparable changes in hubness and clustering coefficient values relative to their respective baseline values. B) A similar task condition effect is seen in the AMY for theta connectivity. C) Changes from baseline in hubness (left) and clustering coefficient (right) characterizing beta connectivity during response selection in the OFC. SERT^−/−^ rats have lower hubness than wildtype counterparts. Hubness and clustering coefficient are higher in case of CS− trials with correct responses. D) Both properties in the AMY are higher in SERT^+/+^ than SERT^−/−^ rats and in the task condition of CS− associated with correct response. E) The OFC of SERT^−/−^ rats display reduced hubness (left) and clustering coefficient (right) for gamma interactions at the time of response selection than the OFC of SERT^+/+^ rats. Only hubness values are significantly more elevated in correct CS− trials in comparison with the other two conditions. F) Lower graph property values are also detected in the AMY of SERT^−/−^ rats in gamma. Task condition affects these values in both genotypes and differentially across genotypes for hubness. Insets in profile plots indicate when an effect is statistically significant (C = task condition, G = genotype, GxC = interaction task condition × genotype) (*n* = 6 SERT^+/+^ rats, *n* = 7 SERT^−/−^ rats).

Finally, we quantified the network approach of task-related synchronization using hubness and clustering coefficient (Figs. 5 and 6). All measures were baseline-subtracted to cancel out the differences already present at baseline (Fig. 4). We calculated one value of each property per animal and per brain area (averaging over electrodes from both hemispheres) for each condition, genotype, and TF synchronization window separately. Hubness and clustering coefficient were not linearly correlated for any of the three synchronization windows (Fig. S4).

For the theta band window around MT, the scatter plot showed apparent clusters based on task condition and brain area but not genotype (Fig. S4A). Specifically, correct CS+ and incorrect CS− trials were characterized in the OFC by relatively high hubness and clustering coefficient, and in the AMY by medium hubness and high clustering coefficient. During correct CS− trials, clustering coefficient and hubness, globally both higher in the OFC than in the AMY, were lower than in the other two conditions and similar across genotypes. We then statistically tested the effects of task condition and genotype in the OFC and AMY separately (Fig. 6A and B). In both brain areas, hubness and clustering coefficient were significantly different across task conditions (Table S4) with correct CS+ and incorrect CS− trials exhibiting significantly higher average values than correct CS− trials, as determined by post-hoc pairwise comparisons. Conversely, neither the genotype nor the interaction condition × genotype had a significant influence (Table S4). Therefore, irrespective of SERT genotype, a highly connected OFC-AMY network characterized the behavioral response made in the side port where reward is a possible outcome.

For the beta and gamma band synchronization windows around RT, the scatter plots showed similarities with an influence of SERT genotype particularly visible in gamma (Fig. S4B and C). Overall, in the two brain areas, a cluster was formed by dots with higher values of hubness and clustering coefficient that corresponded to SERT^+/+^ rats. The task condition also seemed influential in SERT^+/+^ rats with correct CS− trials showing relatively higher hubness, especially in gamma (Fig. S4C). This task condition effect was not as easily discernible in SERT^−/−^ rats. Statistically, the task condition effect was significant in beta for both graph properties and in both brain areas (Fig. 6C and D; Table S4), and in gamma on hubness in both brain areas and on clustering coefficient only in the AMY (Fig. 6E and F; Table S4). Genotype also significantly affected hubness and clustering coefficient in both brain areas and frequency bands, apart from clustering coefficient in the OFC in beta (Table S4). Condition × genotype interaction effects were nonsignificant except for hubness in the AMY in the gamma band (Table S4).

When a significant genotype effect was detected without an interaction effect, post-hoc pairwise comparisons indicated that hubness and clustering coefficient averaged over conditions were lower in SERT^−/−^ than in SERT^+/+^ rats (Fig. 6C–F). Regarding the task condition effect, hubness averaged over genotypes was higher during trials with CS− and correct response than during the other two conditions in the OFC at both frequency bands and in the AMY in beta (Fig. 6C–E, left panels). In the AMY in gamma, it was only higher in SERT^+/+^ rats (Fig. 6F, left panels). Clustering coefficient averaged over genotypes did not vary between task conditions in the OFC in beta (Fig. 6C, right panel; and no task condition effect was seen in the OFC in gamma: Table S4 and Fig. 6E, right panel) but in the AMY, it was higher in the two conditions with CS−, irrespective of their outcome, in both beta and gamma (Fig. 6D and F, right panels). This could suggest that clustering coefficient in the AMY represents a marker of CS discrimination.

Collectively, the graph properties indicated a scarcer functional network at the time of decision-making in the beta and gamma bands in SERT^−/−^ compared to SERT^+/+^ rats.

## Discussion

We found that wildtype and SERT^−/−^ rats learnt the auditory discrimination task in a similar way, although SERT^−/−^ rats showed a nonsignificant trend for a slower acquisition of the association CS−/behavioral response/no reward. Conversely, during LFP recordings once the task was acquired, the accuracy and number of correction trials for CS− trials were in favor of SERT^−/−^ rats. LFP data revealed OFC-AMY synchronization in the beta and gamma bands during response selection. This synchronization was reduced in SERT^−/−^ rats compared to the wildtype littermates, as confirmed by overall reduced hubness and clustering coefficient in the OFC-AMY functional network. In contrast, the theta synchronization observed at the time when a behavioral response was made in the side port associated with rewards, and its corresponding functional network, were similar in both genotypes. Additionally, our data showed variations in the organization of the cross-hemispheric OFC-AMY functional network across frequency bands, with predominantly ipsi- and contralateral interactions in the theta and beta bands and ipsilateral interactions in the gamma band.

### Synchronization dynamics

Beta and gamma synchronization at response selection (Figs. 3 and 5) were stronger for trials during which CS− was played and the rats selected the correct, unrewarded port. In these trials, it is possible that the rats inhibited their preferred response—selecting the side port associated with the reward—and redirected their response to the always unrewarded side port. This might involve OFC neurons encoding directional signals that are highest when behavioral inhibition is most strongly needed, as shown in a variant of the stop-signal task (Bryden and Roesch 2015). Hence, the directional activity of OFC neurons could participate in the task-condition effect of beta and gamma synchronization. Theta synchronization at the time of behavioral response was specific to the side associated with reward delivery, regardless whether a reward was delivered or not (Figs 3, 5, and 6). Theta activity has been shown under similar conditions in humans (Manssuer et al. 2022) and rats (Van Wingerden et al. 2010) and interpreted as a marker of anticipation of reward. Van Wingerden et al. proposed that reward-related theta spike-LFP phase locking in the OFC might promote communication with other brain areas, with AMY being a top candidate. Likewise, we hypothesize that these synchronizations facilitate the interareal transfer of signals of stimulus- or action-informed outcome values encoded by neurons of the OFC and AMY (Sharpe and Schoenbaum 2016).

We also examined the cross-hemispheric network organization between the AMY and OFC, which had weaker contralateral functional connections with increasing frequencies (Figs. 4 and 5), suggesting the engagement of distinct neuronal populations. However, we did not address the question of information transfer direction. Some rare human intracranial LFP studies have shown different directions of information transfer in this network. For example, a greater influence from the AMY to the OFC was reported in a simple choice task (with no cue/action–outcome association) while a bidirectional influence was shown in an emotional task using picture viewing (Jenison 2014; Sonkusare et al. 2023). Although this question remains open in our auditory discrimination task, the established collaboration between the OFC and AMY in outcome-guided decision-making may suggest bidirectional influence (Wassum 2022).

### SERT genotype’s influence on OFC-AMY synchronization

While SERT genotype had no noticeable influence on OFC-AMY theta synchronization in this auditory discrimination task, OFC-AMY beta and gamma synchronization were reduced in SERT^−/−^ rats during response selection (Figs 3, 5, and 6). Evidence of interareal functional connectivity modulations by genetic or pharmacological interventions in the serotonin system have previously been reported with task-, brain region-, and serotonin (5-HT) receptors expression-specific effects. For example, Dzirasa et al. showed increased delta and beta synchronization between the medial prefrontal cortex and AMY in mice genetically deficient for serotonin placed in an open field. The increase in delta synchronization was reversed by chronic fluoxetine (Dzirasa et al. 2013). Theta activity in the AMY has also been shown to be stronger in SERT^−/−^ mice compared to SERT^+/+^ mice during the presentation of a fear-inducing CS predicting foot-shock, while theta synchronization between the AMY and medial prefrontal cortex increased during recall of fear extinction (Narayanan et al. 2011). Moreover, variations in theta and gamma synchronization between the medial prefrontal cortex and hippocampus have been reported in mice after selective pharmacological activation or inhibition of 5-HT receptors (Gener et al. 2019). Optogenetic stimulation of dorsal raphe 5-HT neurons induced a brain-wide modulation of functional connectivity, which correlated with the expression patterns of some 5-HT receptors (Grandjean et al. 2019). It is noteworthy that the expression of several 5-HT receptors, including 5-HT_1A_, 5-HT_2A_, and 5-HT_2C_, differs in SERT^−/−^ animals relative to wildtype controls (Li et al. 2000; Li et al. 2003; Homberg et al. 2008).

The elevated extracellular 5-HT levels of the SERT^−/−^ rats might acutely modulate interareal synchronization, as suggested by these pharmacological and optogenetic studies. However, the effects on functional connectivity between the OFC and AMY in SERT^−/−^ rats might also come from long-lasting consequences of neurodevelopmental alterations, as SERT knockout during early development causes a variety of behavioral and neurobiological effects (Homberg et al. 2010). Behaviorally, SERT^−/−^ rats display delayed development of reflexes, motor function, and social-sensory functions (Kroeze et al. 2016). At the neurobiological level, SERT knockout has been associated with reduced thickness of cortical layer IV and increased neocortical neuronal cell density (Altamura et al. 2007), altered cortical laminar distribution of subtypes of interneurons (Frazer et al. 2015), altered dorsal raphe nucleus/medial prefrontal cortex network formation and altered prefrontal layer identity (Calabrese et al. 2013; Witteveen et al. 2013; Garcia et al. 2019), greater neuronal spine densities in the orbitofrontal cortex and amygdala (Nietzer et al. 2011; Sakakibara et al. 2014), and a reduction in the expression of glutamatergic as well as GABAergic markers, including parvalbumin (PV) in the prefrontal cortex (Guidotti et al. 2012). Interestingly, Canetta et al. found that inhibiting prefrontal PV interneurons during the juvenile and adolescent period in mice resulted in impaired prefrontal task-dependent gamma oscillations and behavioral flexibility in adulthood that could be reversed by the targeted activation of PV interneurons (Canetta et al. 2022). In our study, some alterations in the OFC-AMY functional network were present during the baseline time window (prior to direct trial engagement). Specifically, the ipsilateral OFC-AMY connectivity in the gamma band was nearly absent and average gamma clustering coefficient was reduced in SERT^−/−^ rats compared to SERT^+/+^ rats (Fig. 4). Thus, these baseline genotype differences may reflect constitutive changes in the OFC-AMY network resulting from neurodevelopmental alterations.

Overall, understanding what causes the effect of SERT knockout genotype on OFC-AMY functional connectivity requires further research as it would involve disentangling several potential mechanisms of action, an acute action of elevated extracellular 5-HT levels, long-lasting neurodevelopmental alterations, or subsequent compensatory mechanisms (e.g. altered function of 5-HT receptors autoinhibiting 5-HT neurons; Araragi et al. 2013). Approaches aiming at down-regulating SERT expression (Verheij et al. 2018), modulating 5-HT neurons’ activity through optogenetics or chemogenetics, or pharmacologically blocking or activating 5-HT receptors in adult SERT^−/−^ and SERT^+/+^ animals could be used for such purposes.

### SERT genotype’s influence on behavior

SERT genotype in learning and decision-making involving reward has primarily been implicated in adaptive behavior, for example, during outcome devaluation or reversal learning (Brigman et al. 2010; Nonkes et al. 2010; Nonkes et al. 2013). Similarly, induced variations in 5-HT availability or 5-HT receptors binding, at least within the OFC, influence reversal learning and outcome devaluation (Bari et al. 2010; Boulougouris and Robbins 2010; West et al. 2013; Alsiö et al. 2021; Ohmura et al. 2021; Hyun et al. 2023). In these studies, the initial discrimination learning phase preceding reversal learning was not affected by serotonergic drug manipulations. Our data align with these observations, as neither the response accuracy nor the number of corrections for CS+ trials (associated with reward obtention) was influenced by SERT genotype (Figs 1 and 2). Considering that DRN 5-HT neuron activation slows down locomotor activity during exploration but not during reward seeking (Correia et al. 2017), it is also noteworthy that, for CS+ trials, both genotypes had similar RD and that SERT^−/−^ rats’ MD reduces to converge with that of SERT^+/+^ rats during the training phase (Figs 1 and 2).

Our auditory discrimination task also tested decision-making in the absence of reward, for which little is known in SERT knockout rats or mice. In the learning phase, SERT^−/−^ rats tended to acquire the association between CS− and the correct behavioral response in the unrewarded side port later than SERT^+/+^ rats. Intriguingly, we observed opposite trends while recording LFPs after successful task acquisition. SERT^−/−^ rats showed then trends of higher accuracy and decreased number of corrections for CS− trials together with longer RDs for correct CS− trials, suggesting they opted for accuracy rather than speed (Fig. 2). First, none of these results passed statistical significance and future research should confirm these trends with increased power. Second, the discrepancy between the learning and maintenance phases of the task regarding CS− trials might reveal different capacities of SERT^+/+^ and SERT^−/−^ rats to face increasing task difficulty (in this case, likely due to the weight or limitation of movement of the implant). In fact, several indicators seen in both genotypes suggest the task is more difficult, e.g. requires more motivation or patience, during LFP recordings compared to before surgery: (i) the accuracy for CS− trials is lower (while CS+ accuracy is >80% suggesting the task is remembered), (ii) MDs are increased in all types of trials, and (iii) ITIs are also increased (Figs. 1 and 2). Interestingly, serotonin has been linked to promoting patience and recently active persistence (Lottem et al. 2018). We can thus hypothesize that SERT^−/−^ rats, through elevated 5-HT extracellular levels, would be more willing to perform an unrewarded action in order to obtain later a reward, and thereby more resilient than SERT^+/+^ rats to the decreased value of CS− trials linked to increased task difficulty. The hypothesis is also interesting as a potential link between behavior and LFP data. At the time of decision-making, correct CS− trials entail greater gamma synchronization between the OFC and AMY than other task conditions, mainly in SERT^+/+^ rats (Fig. 3). Since gamma oscillations have been shown to be associated with high energy demand (Kann et al. 2011), it is tempting to speculate that correctly performing CS− trials during LFP recordings might have a lower energetic cost in SERT^−/−^ rats than in SERT^+/+^ rats.

### Limitations

A first limitation of our study is the lack of power to demonstrate statistical significance for findings of the behavioral data, limiting their interpretation. Another limitation is that we studied male rats only, hindering generalizability to females. Given that differences among sexes have been reported in some decision-making tasks in rodents (Orsini and Setlow 2017), OFC-AMY synchronization should also be assessed in females in the future.

## Conclusion

The primary result of this study is the decreased functional connectivity in beta and gamma between the OFC and AMY at the time of response selection in SERT^−/−^ rats. This finding suggests SERT genotype may regulate behavior by modulating interareal functional networks. Future studies in this direction have the potential to reveal the neural underpinnings of SERT-genotype dependent changes in sensitivity and responsivity to environmental stimuli (Homberg and Jagiellowicz 2022). Furthermore, as SERT knockout rats model the short allelic variant of the human serotonin transporter–linked polymorphic region at least regarding stress sensitivity (Schipper et al. 2019) and likely proneness to stress-related psychiatric disorders such as anxiety-related disorders and depression (Caspi et al. 2010), the present findings help to understand the neurobiological underpinnings of risk to these disorders.

## Supplementary Material

Supplementary_material_Boillot_et_al_final_bhae334
