# Supplementary material for: Serotonin transporter knockout in rats reduces beta- and gamma-band functional connectivity between the orbitofrontal cortex and amygdala during auditory discrimination
Source: Cereb Cortex. 2024 Aug 11;34(8):bhae334. doi: 10.1093/cercor/bhae334 (PMC11317204; doi:10.1093/cercor/bhae334)
Supplement: Supplementary_material_Boillot_et_al_final_bhae334 [file supplementary_material_boillot_et_al_final_bhae334.docx]

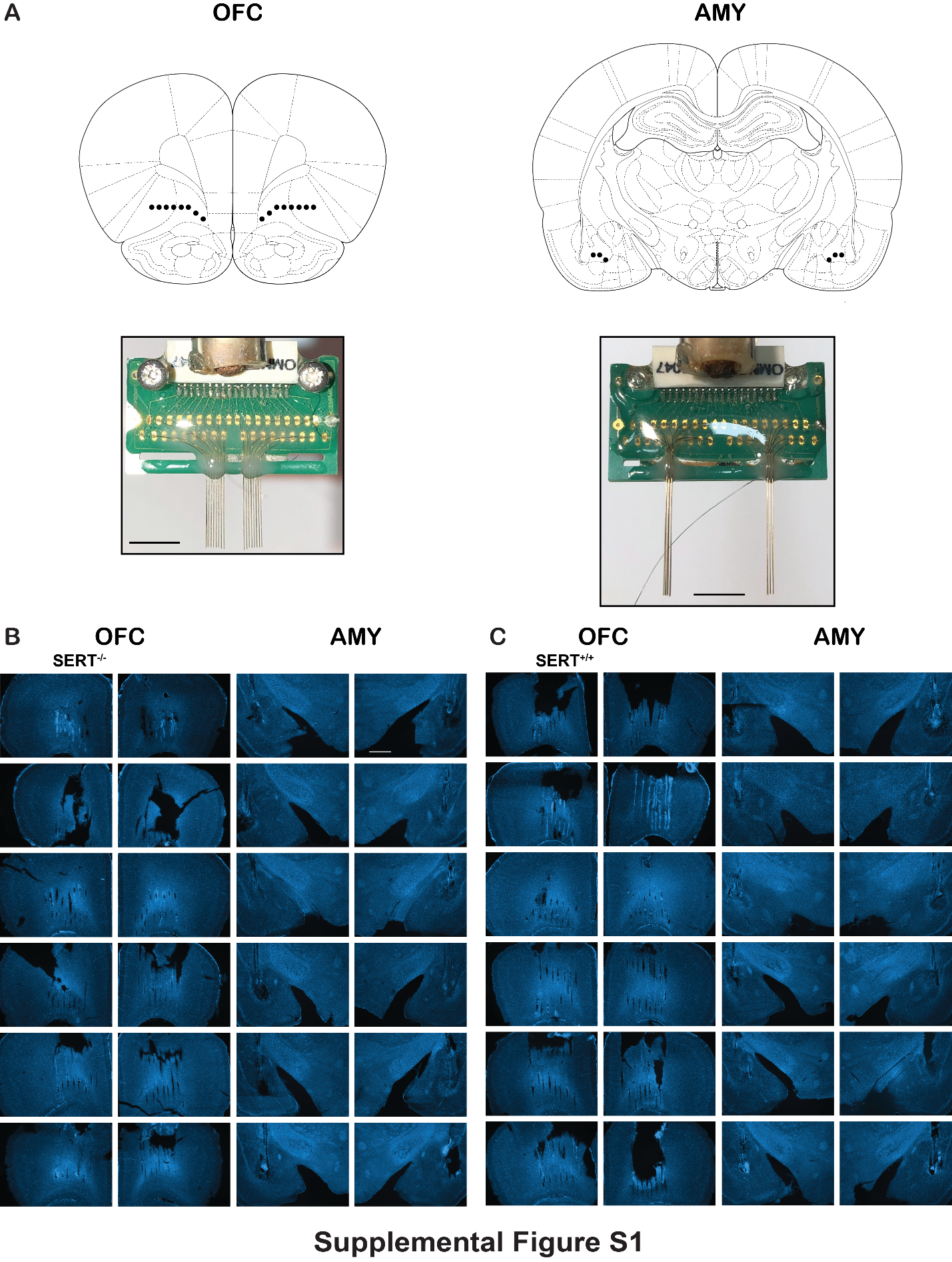


**Figure S1. A,** Top left: Theoretical location of one out of two rows of the LFP electrodes in the OFC of both hemispheres. Schematic coronal section of a rat brain +3.7 mm from bregma. Bottom left: Example of an electrode array implanted in the OFC of both hemispheres. Only the tungsten wires (lower part) are lowered into the brain. Top right: Theoretical location of one out of five rows of the LFP electrodes in the AMY of both hemispheres. Schematic coronal section of a rat brain -2.6 mm from bregma. Bottom right: Example of an electrode array implanted in the AMY of both hemispheres. **B-C,** Representative images of DAPI-stained brain slices with visible electrode tracks of SERT^-/-^ rats (**B**) and SERT^+/+^ rats (**C**). Each row represents one animal. The two columns on the left and right include the OFC and the AMY of each hemisphere, respectively. Images are shown for each animal used in the LFP analysis, except for one SERT^-/-^ rat represented in Figure 2A. Scale bar (in white, top right image) = 1 cm; identical for all images.


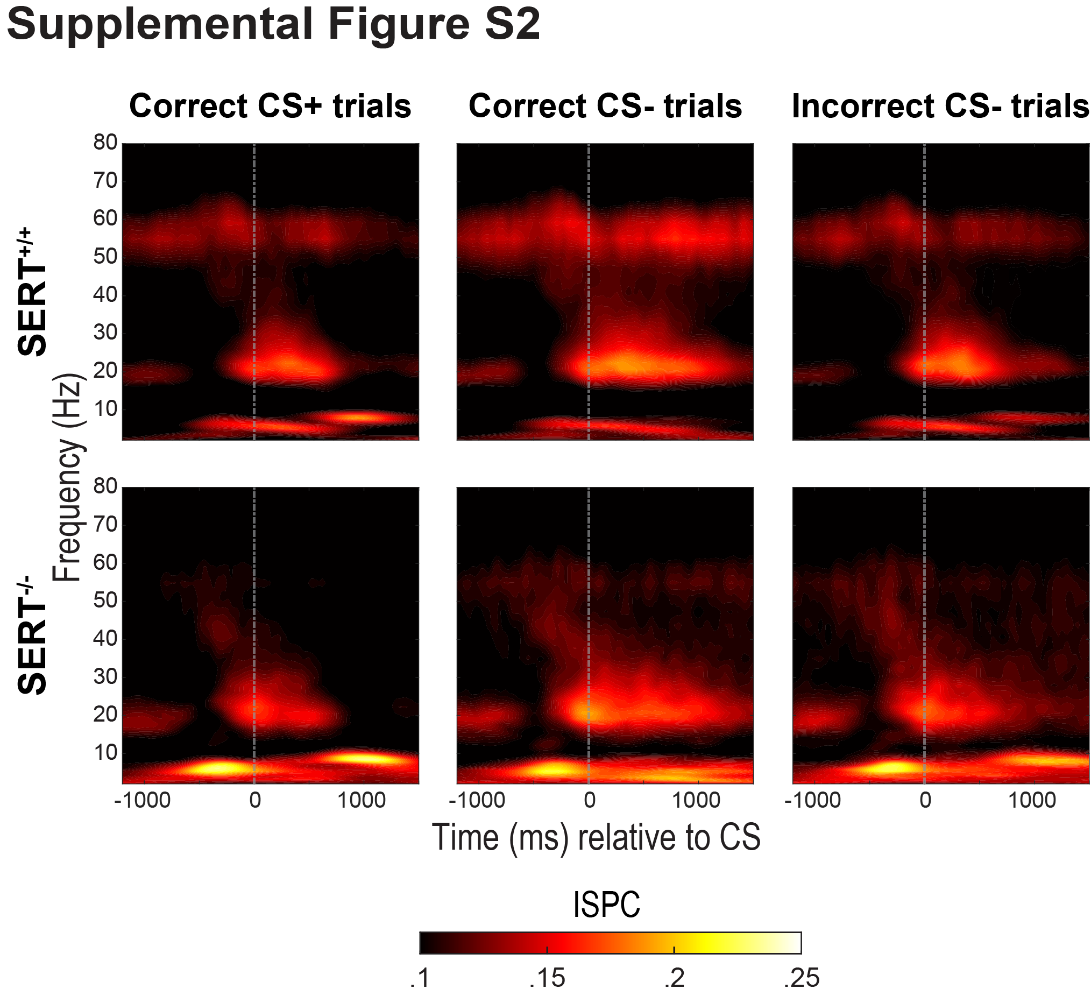


**Figure S2.** Time-frequency representations of the same ISPC changes as in Fig. 3A (top) when time series are locked to CS onset but averaged over trials within a task condition and over animals within a genotype.

**
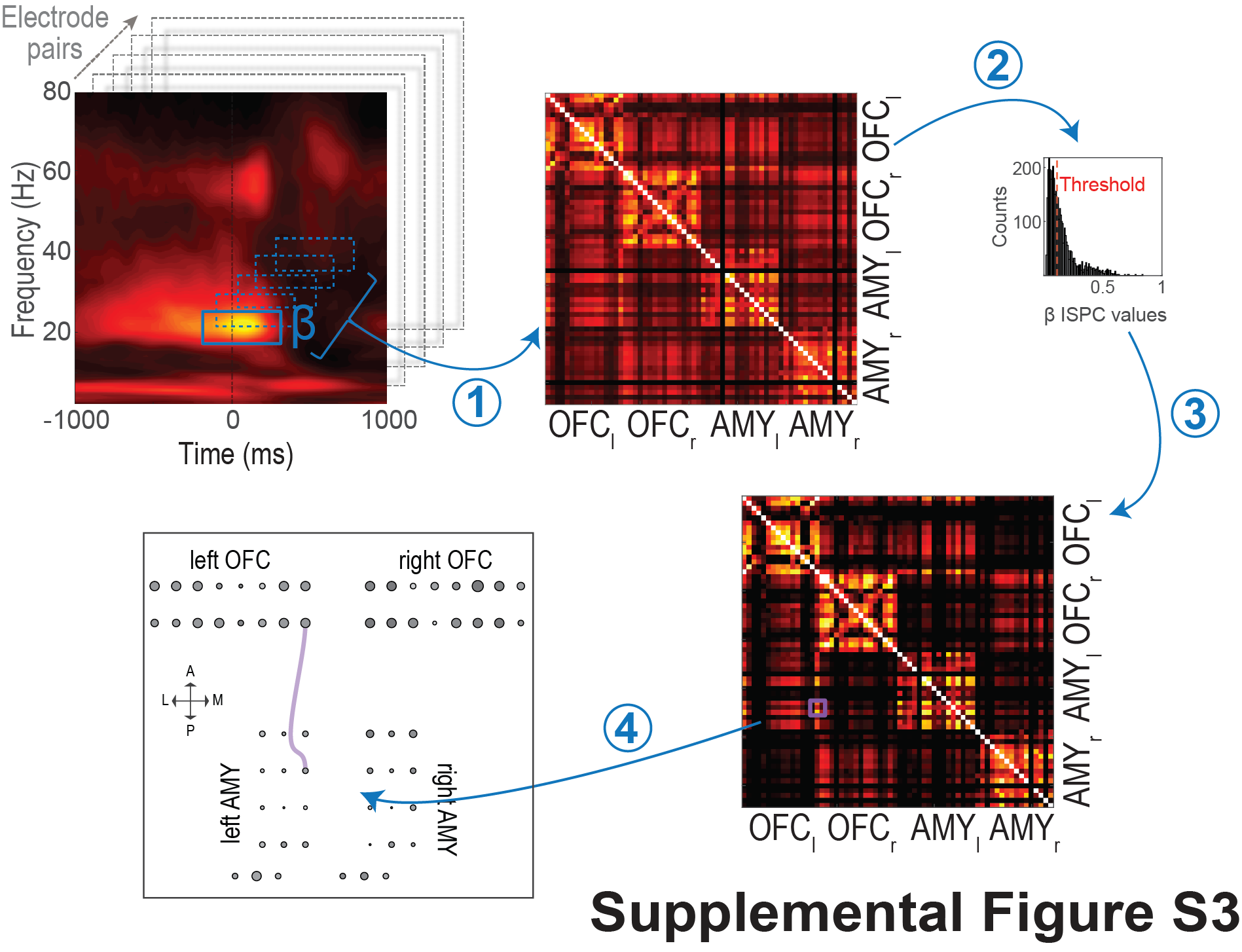
**

**Figure S3.** Schematic representation of the four steps from the ISPC time-frequency analysis to the graphical visualization of the cross-hemispheric OFC-AMY functional network. Step 1: an ‘all-to-all’ connectivity matrix is generated by averaging ISPC values over time and frequency within a selected window (here, beta) for every pair of electrodes. This ‘all-to-all’ synchronization is thresholded at the median to keep task-related information (step 2), resulting in a matrix where all synchronization values below the threshold are set to zero (step 3). Step 4: The suprathreshold matrix is converted into a network graph where each node depicts an electrode (8 columns x 2 rows in each OFC, 3 columns x 5 rows in each AMY) and the curves represent the suprathreshold connectivity between electrode pairs. For illustration purposes, we here only draw one purple curve representing one pixel of the matrix.


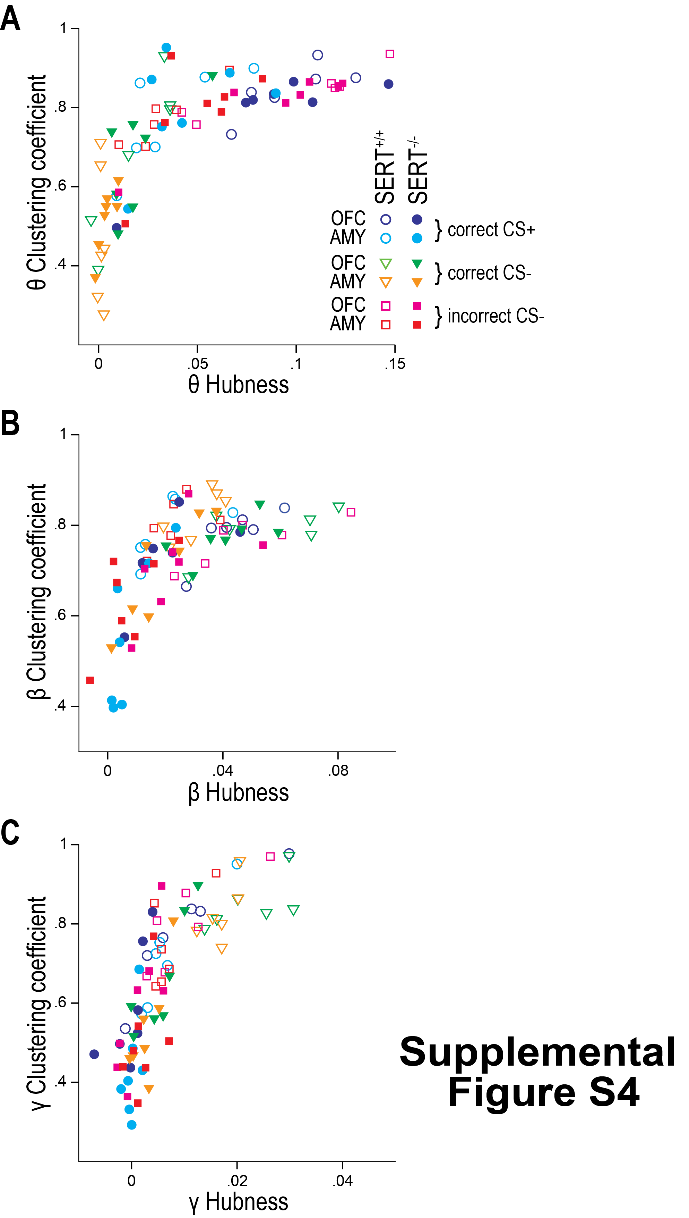


**Figure S4.** Hubness and clustering coefficient provide non-redundant information about the cross-hemispheric OFC-AMY network during task-relevant functional interactions. Graphic representation of clustering coefficient as a function of hubness during theta connectivity around MT in **A**, during beta connectivity around RT in **B**, and during gamma connectivity around RT in **C**. Each marker represents the average for one animal.

**Table S1.**  Degrees of freedom, F values and p values from ANOVAs of the effects of training, SERT genotype and their interaction on behavioral parameters during task acquisition.

**Table S2.** Degrees of freedom, F values and p values from ANOVAs of the effects of training, SERT genotype and their interaction on behavioral parameters during LFP recordings.

**Table S3.** Degrees of freedom, F values and p values from ANOVAs of the effects of the frequency band, SERT genotype and their interaction on hubness and clustering coefficient in the OFC and AMY during baseline.

**Table S4.** Degrees of freedom, F values and p values from ANOVAs of the effects of the task condition, SERT genotype and their interaction on hubness and clustering coefficient in the OFC and AMY in theta, beta and gamma synchronization.
